# Manganese(II) ions suppress the transcription of the citrate exporter encoding gene *cexA* in *Aspergillus niger*

**DOI:** 10.3389/fbioe.2023.1292337

**Published:** 2023-11-22

**Authors:** Aline Reinfurt, Susanne Fritsche, Vivien Bíró, Alexandra Márton, Valeria Ellena, Erzsébet Fekete, Erzsébet Sándor, Levente Karaffa, Matthias G. Steiger

**Affiliations:** ^1^ Research Group Biochemistry, Institute of Chemical, Environmental and Bioscience Engineering, TU Wien, Vienna, Austria; ^2^ Austrian Centre of Industrial Biotechnology (ACIB GmbH), Vienna, Austria; ^3^ Department of Biochemical Engineering, Faculty of Science & Technology, University of Debrecen, Debrecen, Hungary; ^4^ Juhász-Nagy Pál Doctoral School of Biology and Environmental Sciences, University of Debrecen, Debrecen, Hungary; ^5^ Institute of Food Science, Faculty of Agricultural and Food Science and Environmental Management, University of Debrecen, Debrecen, Hungary

**Keywords:** citric acid transporter, citric acid overflow, manganese effect, Aspergillus niger, cexA regulation, metal ions, major facilitator superfamily

## Abstract

*Aspergillus niger* is an important filamentous fungus used for the industrial production of citric acid. One of the most important factors that affect citric acid production is the concentration of manganese(II) ions present in the culture broth. Under manganese(II)-limiting conditions, the fungus develops a pellet-like morphology that is crucial for high citric acid accumulation. The impact of manganese(II) ions on the transcription of the major citrate exporter encoding gene *cexA* was studied under manganese(II)-deficient and -sufficient conditions. Furthermore, citric acid production was analyzed in overexpression mutant strains of *cexA* in the presence and absence of manganese(II) ions, and the influence of CexA on fungal morphology was investigated by microscopy. Transcriptional upregulation of *cexA* in the absence of manganese(II) ions was observed and, by decoupling *cexA* expression from the native promoter system, it was possible to secrete more citric acid even in the presence of manganese. This effect was shown for both an inducible and a constitutive overexpression of *cexA*. Furthermore, it was found that the presence of CexA influences fungal morphology and promotes a more branched phenotype. According to this study, manganese(II) ions suppress transcription of the citrate exporter *cexA* in *Aspergillus niger*, causing citric acid secretion to decrease.

## 1 Introduction

Citric acid (2-hydroxy-propane-1,2,3-tricarboxylic acid) is a weak organic acid occurring as an intermediate of the tricarboxylic acid cycle in aerobic organisms and can be secreted by fungi like *A. niger*. Due to its properties, this organic acid has a variety of industrial applications ranging from foods and beverages to detergents and pharmaceuticals (Behera, 2020; Reena et al., 2022). The vast majority of citric acid is manufactured by large-scale industrial fermentations employing the filamentous fungus *Aspergillus niger* (Karaffa et al., 2001; Show et al., 2015; Behera, 2020). In comparison to other hosts, the production of citric acid with *A. niger* delivers exceptionally high yields (Show et al., 2015): up to 95 kg of citric acid per 100 kg of sugar can be obtained (Karaffa and Kubicek, 2003; Karaffa and Kubicek, 2019). In order to trigger a citric acid overflow metabolism and reach such high yields, specific requirements must be met within the cultivation medium and process (Karaffa and Kubicek, 2003): high citric acid production occurs in the concomitant presence of high sugar concentrations, phosphate limitation, and high dissolved oxygen supply. Additionally, metal ions, mainly manganese(II) (Kisser et al., 1980; Meixner et al., 1985; Netik et al., 1997; Karaffa et al., 2015), furthermore referred to as “manganese”, and iron(II) (Odoni et al., 2017), must be in limiting concentrations to achieve high citric acid yields (Karaffa et al., 2021). In the absence of the above-described culture conditions, the fungus displays filamentous morphology and is not able to produce high amounts of citric acid. On the contrary, under these conditions, the hyphae of the fungus become swollen and shortened, and a pellet-like morphology is developed. The pellet morphology is crucial for high citric acid secretion (Clark et al., 1966; Kisser et al., 1980; Karaffa and Kubicek, 2003). This phenotype, contrary to the hairy and filamentous morphology, allows for an increased oxygen transfer into the liquid and subsequently to the cells, ensuring a high oxygen supply (Kisser et al., 1980; Karaffa and Kubicek, 2003; Tong et al., 2019). It was previously observed that drastic morphological changes occur by simply changing the concentration of manganese in the medium. In the presence of sufficient manganese concentration (>5 μg/L), the fungus grows filamentous and the final volumetric yield can be reduced by up to 20%. Manganese-deficiency (<5 μg/L) transforms the filamentous hyphal morphology to one dominated by shortened, swollen branches on the micro-morphology level and small compact (<0.5 mm diameter) pellets on the macro-morphology level (Detroy and Ciegler, 1971; Gyamerah, 1995). This transformation is accompanied by increased cell/hyphae diameter and reduced pellet size (Paul et al., 1994; Karaffa et al., 1997; El-Sabbagh et al., 2008). High (>75%) specific molar yields (Yp/s) occur only when cultures are characterized by such morphology (henceforth referred to as “overflow-associated morphology”) (Sándor et al., 2021).

Manganese is an essential metal ion needed for several physiological processes. It acts as a cofactor for a set of enzymes interacting with nucleotides, such as RNA polymerases, some kinases, and dehydrogenases (Crowley et al., 2000; Culotta et al., 2005). It is also relevant for several sugar transferases of the Golgi apparatus (Karaffa et al., 2021). Manganese in yeast and filamentous fungi acts as a key cofactor for the manganese-dependent mitochondrial superoxide dismutase (MnSOD) (Reddi et al., 2009; Lambou et al., 2010). Superoxide dismutases (SODs) are metalloenzymes responsible for protecting fungi against reactive oxygen species (ROS) (Del Valle and Scheckhuber, 2022). By detoxifying superoxide anions, they play a crucial role in protecting against oxidative stress. Under manganese-deficiency, MnSOD is severely inhibited leading to higher oxidative stress, which results in increased protein turnover (Ma et al., 1985), and altered composition of the plasma membrane (Meixner et al., 1985) and the cell wall (Kisser et al., 1980). This might be connected with the development of a highly branched mycelium with thickened cell walls and thick, swollen hyphae leading to the pellet-like morphology (Karaffa et al., 2021). Furthermore, it was shown that secreted citrate can only be taken up when bound to manganese. Therefore, under manganese-limiting conditions, secreted citrate cannot be imported again in high amounts, underlining the importance of manganese-deficiency in citric acid overflow (Netik et al., 1997). The uptake of manganese in *A. niger* is controlled by the manganese transporter DmtA (Fejes et al., 2020). Upon deletion of the manganese transporter encoding gene *dmtA*, manganese uptake is strongly limited, and high citric acid titers (100 g/L) can be achieved even at manganese concentrations of 100 μg/L (Fejes et al., 2020). This transporter thus represents an engineering target for simulating manganese-deficient conditions in a manganese-sufficient environment (Fejes et al., 2020). Secretion of citrate into the culture broth occurs via the main citrate exporter CexA (Steiger et al., 2019). This transmembrane protein belongs to the major facilitator superfamily. One known regulator of *cexA* is the methyltransferase LaeA (Niu et al., 2016; Kadooka et al., 2020). The control mechanism of LaeA is based on altering the methylation levels of the histones H3K4 and H3K9 at the *cexA* promoter, thus regulating the accessibility of the RNA polymerases to the gene (Kadooka et al., 2020). Due to the crucial role played by manganese on the secretion of citrate, the effect of manganese on the transporter *cexA* is investigated in this study.

## 2 Materials and methods

### 2.1 Strains

All strains used in this study are listed and described in Table 1.

**TABLE 1:** Strains and their relevant genotype and function.

Name	Relevant genotype	Function	Source
ATCC 1015	ATCC 1015	Parental strain/Wild-type	American Type Culture Collection (Andersen et al., 2011)
cE-*cexA*	pyrG::P_*mbfA*_:*cexA*:TtrpC, pyrG^+^, ATCC 1015 background	Constitutive *cexA* overexpression	Steiger et al. (2019)
iE-*cexA*	pyrG::P_tet-on_:*cexA*:TtrpC, pyrG^+^, ATCC 1015 background	Inducible *cexA* overexpression	Steiger et al. (2019)
D-*cexA*	Disruption of *cexA* coding sequence by a SNP, ATCC 1015 background	loss-of-function mutation of *cexA*	Steiger et al. (2019)

### 2.2 Media and cultivation conditions

All chemicals used in this study were analytical grade and were purchased from Carl Roth GmbH + Co. KG, Austria, or Sigma Aldrich, Austria, and Sigma Aldrich, Hungary.

All fungal strains were maintained at 30 °C for 5 days on minimal medium (MM) (Barratt et al., 1965) before harvesting the conidia for cultivation. Conidia were harvested using 0.1% Tween20 and washed twice (centrifugation: 5,000 rpm, 10 min) with the same solution before resuspending in 0.1% Tween20. Conidial concentration was determined using a Thoma chamber.

#### 2.2.1 Shake flask cultivations

Glassware and plasticware used for shake flask cultivations were treated overnight with 1.5 M HCl. Solutions and media were prepared with manganese-free water, as described in 2.2.3. The solid and chemically defined liquid media were prepared as previously described (Fekete et al., 2022), with the exception of the d-glucose concentration in the liquid medium, which was 140 g/L. Baffled 1 L flasks with 100 mL medium were used for cultivation. Manganese-deficient and manganese-sufficient culture conditions were tested. Manganese was added in the form of MnCl_2_*4H_2_O. All cultivations were performed in biological triplicates. Samples for RNA extraction and HPLC analysis were taken after 24, 48, and 72 h. Mycelial samples for RNA extraction were first washed with Milli-Q water, paper-dried, and stored in 500 µL RNAzol RT reagent (Sigma-Aldrich, Austria) at −80°C for 4 days before RNA extraction. The supernatant of the samples was analyzed by HPLC.

#### 2.2.2 Microtiter plate (MTP) cultivations

MTP cultivations were performed in 96-well plates in a volume of 100 µL. An adapted version of Vogel’s minimal medium (VM) for high citric acid production was used for MTP cultivations. The medium was supplemented with 20% d-glucose and 0.5% citric acid, the remaining composition was as described in Steiger et al. (2019). The initial pH of the medium was set to 5.6. Different amounts of MnCl_2_*4H_2_O (final manganese(II)-concentrations: 0 μg/L, 5 μg/L, and 100 μg/L) were added, depending on the culture condition to be tested. All cultivations were performed without and with doxycycline (4 μg/mL) in order to induce *cexA* overexpression in the iE-*cexA* strain. For each condition, 10 mL VM were inoculated with 10^8^ conidia/L of the strain of interest. Cultivations were performed at 30 C, 85% humidity, and 240 rpm for 72 h in a Minitron incubation shaker (Infors HT Minitron, Bartelt, Austria, 5 cm amplitude). After cultivation, the supernatant was collected by filtration and analyzed by HPLC.

#### 2.2.3 Bioreactor cultivations

Glassware and plasticware used for bioreactor cultivation were treated overnight with 1.5 M HCl to remove all traces of manganese. Solutions and media were prepared with manganese-free water obtained by treating Milli-Q water (Arium Mini, Sartorius, Germany) by column ion exchange chromatography (AmberChrom 50Wx8 100–200 (H), Thermo Scientific, Germany).

Solid cultures on minimal medium plates and cultivations in chemically defined liquid medium (pre-culture and bioreactor culture) were performed as previously described using the same media (Fekete et al., 2022). For pre-cultures, 100 mL of medium were inoculated with 10^9^ conidia/L in six 500 mL Erlenmeyer flasks and grown for 24–26 h. Pre-cultures were pooled together and added into the bioreactor (Sartorius Biostat B, Göttingen, Germany) containing the sterile culture medium, reaching a final volume of 5 L. Two bioreactors were operated simultaneously. Cultivation A was performed under manganese-deficient conditions (no supplemented manganese), and cultivation B was performed in sufficient manganese conditions (100 μg/L supplemented manganese in the form of MnCl_2_*4 H_2_O). The bioreactor cultivations were performed without pH control, 900 rpm stirring speed (except for manganese-free cultivation, where stirring speed was reduced to 700 rpm after 200 h), 3 L/min aeration (air), and a constant temperature of 30 C. Samples were taken every 24 h and filtered before HPLC analysis. For dry cell weight (DCW) analysis during bioreactor cultivations, 5 mL of sample were filtered onto a pre-weighted filter paper, dried for 1 h at 70 C, and weighed.

### 2.3 RNA extraction

400 µL RNAzol RT reagent (Sigma-Aldrich, Austria) as well as 0.25 g 0.1 mm ∅ and 1 mm ∅ glass beads were added to the mycelium samples prior to sample processing. Cell disruption was achieved using a bead ruptor (MP Fastprep-24, Fisher Scientific, Germany) for three cycles of 20 s at 6 m/s. The samples were placed on ice for 1 min between each cycle. After cell disruption, the samples were centrifuged at 12,000 g for 5 min at room temperature. The supernatant was used for further RNA extraction using the innuPREP RNA Mini Kit 2.0 (Analytic Jena GmbH, Germany), according to the manufacturer’s instructions. The concentration and the quality of the extracted RNA were evaluated using a UV-Vis spectrophotometer (NanoDrop one^c^ Thermo Scientific, Germany) and agarose gel analysis (1.5% gel, 130 V, 20 min) prior to cDNA synthesis.

### 2.4 DNase digest and cDNA synthesis

DNase digest was performed on the extracted and purified RNA samples using the DNase I Reaction Kit (NEB, United Kingdom), by incubating 1 µg of RNA for 1 h at 37 C and subsequent heat inactivation of the enzyme.

cDNA synthesis was performed with 1 µg of DNase I-treated RNA sample using the Luna Script RT SuperMix Kit (NEB, United Kingdom).

### 2.5 qPCR analysis

Quantitative PCR (qPCR) was performed on a Rotor-Gene Q series, (Qiagen, Germany) with cDNA samples diluted 1:10 in nuclease-free ultrapure water using the Luna Universal qPCR Master Mix (NEB, United Kingdom). The primers for the analysis are listed in Table 2. The suggested cycler program was used for all runs. The log2fold changes of gene expression between the control conditions (manganese-sufficient) and the samples (manganese-sufficiency) were calculated according to the previously published method by Pfaffl, 2001. The housekeeping genes *actA* and *sarA* were used as reference genes.

**TABLE 2:** Primers used for qPCR analysis.

Gene to be detected	Forward primer 5′-3′	Reverse primer 5′-3′
*actA*	GGT​CTG​GAG​AGC​GGT​GGT​AT	GGT​CTG​GAG​AGC​GGT​GGT​AT
*sarA*	GAG​ACA​GGG​ATA​CGG​TGA​AG	GAG​ACA​GGG​ATA​CGG​TGA​AG
*cexA*	CTA​GGC​AAT​GGC​TTT​GGA​TGT​ATG​TC	GGA​AGT​CGG​GGT​GTG​ATT​TCA​G

### 2.6 HPLC analysis

Supernatants of the MTP and shake flask cultivations were analyzed by HPLC (HPLC, Shimadzu, LC-20AD series, Japan) equipped with a Bio-Rad Aminex HPX-87H^+^ column (Bio-Rad Inc., United States) and operated with 5 mM H_2_SO_4_ as mobile phase.

Bioreactor samples were analyzed by HPLC (HPLC, Agilent Technologies 1.260 Infinity, United States) equipped with a Bio-Rad Aminex HPX-87H^+^ (Bio-Rad Aminex HPX-87H^+^, Bio-Rad Inc., United States) and using 10 mM H_2_SO_4_ as mobile phase. In all samples, citric acid and d-glucose were determined based on a calibration curve using pure standards.

### 2.7 Morphological analysis

Fungal morphology was characterized as described earlier (Sándor et al., 2021). Three distinct fungal forms were defined: (a) the swollen hyphal fragments, (b) the filamentous hyphae and (c) the mycelial pellets (Bartoshevich et al., 1990; Paul and Thomas, 1998). The morphology of the cells over the cultivation course was investigated microscopically with an Axio-Vision AC quantitative image analyzer system. To increase contrast and visibility, lactophenol cotton blue (Fluka Chemie, Buch, Switzerland) was added to the medium samples in a final concentration of 10% (v/v). Stained samples were analyzed with a Zeiss AxioImager phase-contrast microscope equipped with AxioCam MRc5 camera. Microscopic pictures (at least two at each time point) were taken regularly every 24 h from the cultures and the number of filamentous and round cells was recorded. Round cells were defined as those cells with a length/width ratio <1.5. Among those, giant round cells were defined as round cells with a cell diameter >2x the hyphal diameter (Supplementary Figure S5).

### 2.8 Digital holotomographic microscopy

*Aspergillus niger* ATCC 1015, iE-*cexA, and* D-*cexA* were analyzed. After conidia harvesting and counting, the strains were inoculated in 20 mL chemically defined liquid medium described in (Fekete et al., 2022), at a concentration of 5 × 10^3^ conidia/mL and with 4 μg/mL doxycycline, if required. Microscope coverslips were mounted on the bottom of Petri dishes. After 12 h at 30 °C, coverslips were mounted on microscope slides for immediate 3D digital holotomographic microscopy (DHTM). 4D live-cell DHTM of *A. niger* ATCC 1015 was performed by inoculating 3.5 × 10^5^ conidia/mL in 500 µL manganese-deficient chemically defined medium or 500 µL manganese-sufficient chemically defined medium. After seeding in a 35 mm IbiTreat polymer bottom µ-Dish (Ibidi GmbH, Munich, Germany), conidia were settled for 1 hour, then the medium was discarded, and the dish was washed three times with fresh medium and subsequently filled with 1.5 mL of the respective medium. Digital holotomographic microscopy (DHTM) for pre-incubated strains and for live-cell imaging was performed using the 3D Cell Explorer-fluo microscope (Nanolive SA, Ecublens, Switzerland). For long-term live cell imaging, the 35 mm dish was placed in a stage-top incubator system and a constant temperature of 30 °C was maintained by a temperature controller (Okolab, Italy). Water reservoirs were placed into the incubation chamber to protect the culture from evaporation. 4D RI tomograms were obtained at a temporal resolution of one frame every 2 min.

### 2.9 Image processing and analysis

**FIGURE 1:**
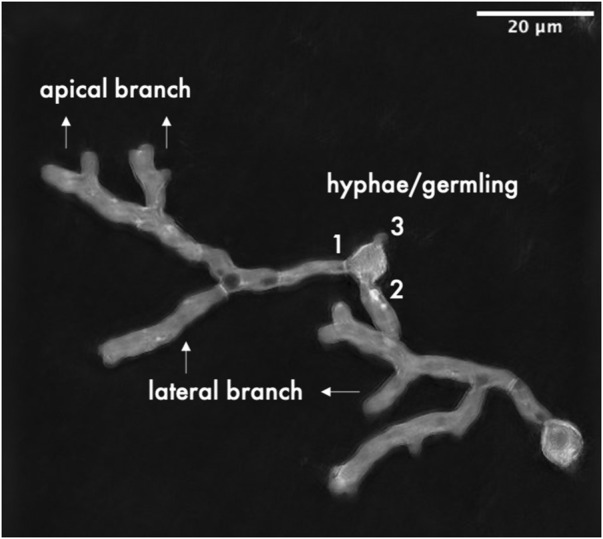
Graphical summary of quantified morphological parameters.

3D and 4D stacks of DHTM were cropped in the z-axis to include only slices that contained specimens. TIFF files were imported into FIJI software version 2.9.0/1.53. t (Schindelin et al., 2012). Stack-projection was done by applying maximum projection of 3D and 4D time-lapse acquisitions. Determination of morphological analysis included counting of hyphae per germling, and the number of lateral and apical branches (Figure 1). Mean values were compared using Student’s t-test whenever indicated, and the significance level for determining statistical significance was set at *p* < 0.05.

## 3 Results and discussion

### 3.1 The transcription of *cexA* is upregulated in the absence of manganese

The presence of manganese in the cultivation was shown to have a negative effect on citric acid secretion (Kisser et al., 1980; Karaffa and Kubicek, 2003; Karaffa et al., 2021). Furthermore, it was found that *cexA* is essential for citric acid secretion (Odoni et al., 2019; Steiger et al., 2019), thus we hypothesized that manganese could have an influence on the transcription of *cexA*. To test this, transcript levels of *cexA* were measured by RT-qPCR in the citric acid-producing parental strain ATCC 1015 cultivated under manganese-sufficient and -deficient conditions.

**FIGURE 2:**
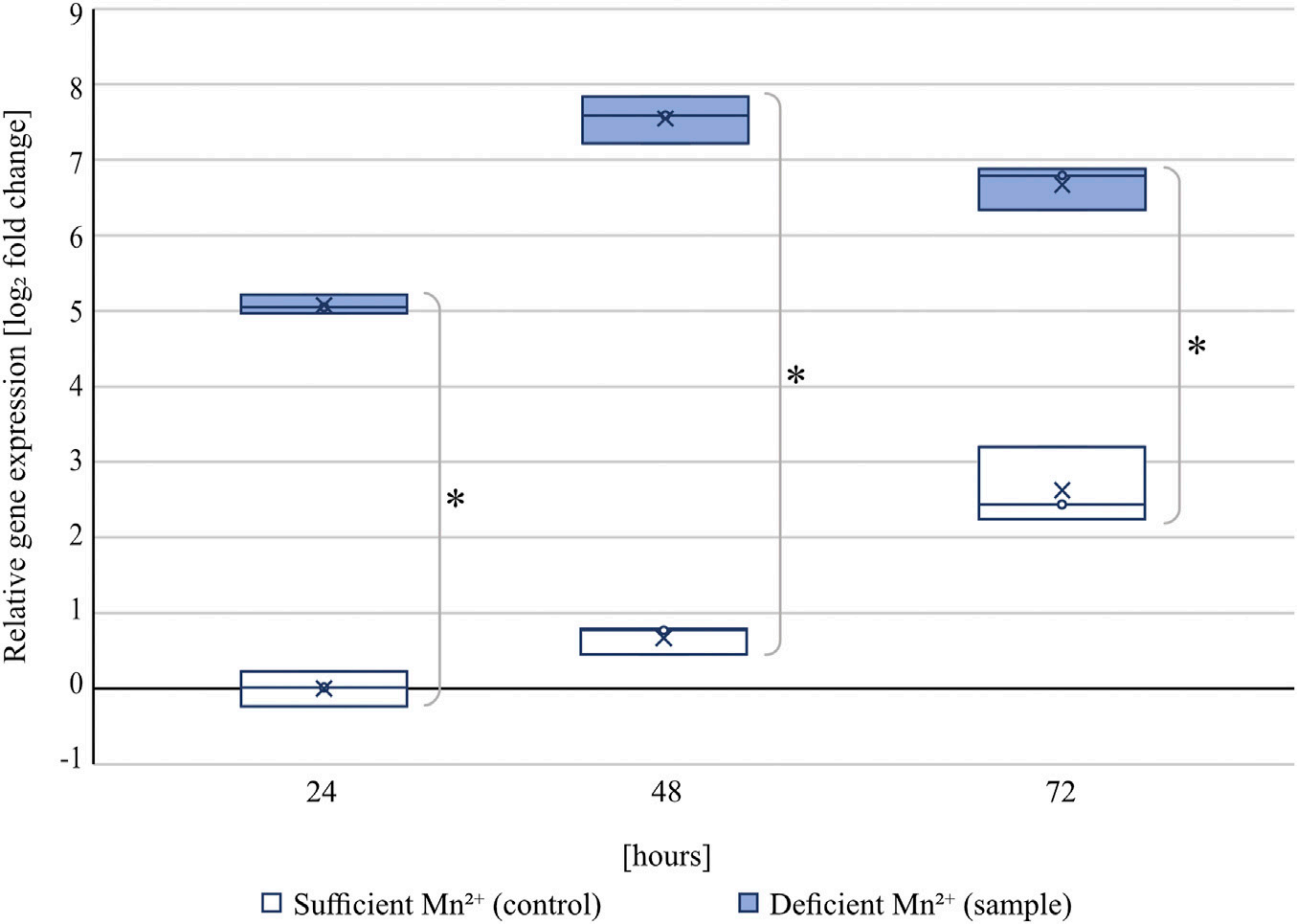
Relative transcript levels of *cexA* under manganese-sufficient (white boxes) and manganese-deficient (light blue boxes) conditions of strain ATCC 1015 at 24, 48, and 72 h cultivated in shake flasks. Within the boxes, the line represents the median and the cross the arithmetic mean of three independent biological replicates. Samples were referenced to the 24 h time point under manganese-sufficient conditions. Significant differences (*p* < 0.01) determined by t-test (n = 3) are highlighted by an asterisk.

At all tested time points, the transcript levels of the citrate exporter *cexA* were significantly upregulated under manganese-deficient conditions compared to manganese-sufficient conditions (Figure 2).

At 24 h, the transcript of *cexA* was 34-fold upregulated under manganese-deficiency compared to manganese-sufficiency, which increased further to a fold change of 187 after 48 h (4.6 g/L citric acid in manganese-deficiency; 0 g/L citric acid in manganese-sufficiency) and 102 after 72 h (12.9 g/L citric acid in manganese-deficiency; 0.15 g/L citric acid in manganese-sufficiency). For both cultivation conditions, *cexA* transcription was observed to increase with time, which is in agreement with the onset of citric acid formation between 24 and 72 h. The transcript levels under manganese-sufficient conditions are still below the transcript levels measured under all manganese-deficient conditions investigated, increasing by about 6 fold after 72 h. This data shows that *cexA* is strongly induced in the absence of manganese(II) ions.

### 3.2 Deregulation of the transcription of *cexA* can counteract the negative effect of manganese on citric acid secretion

**FIGURE 3:**
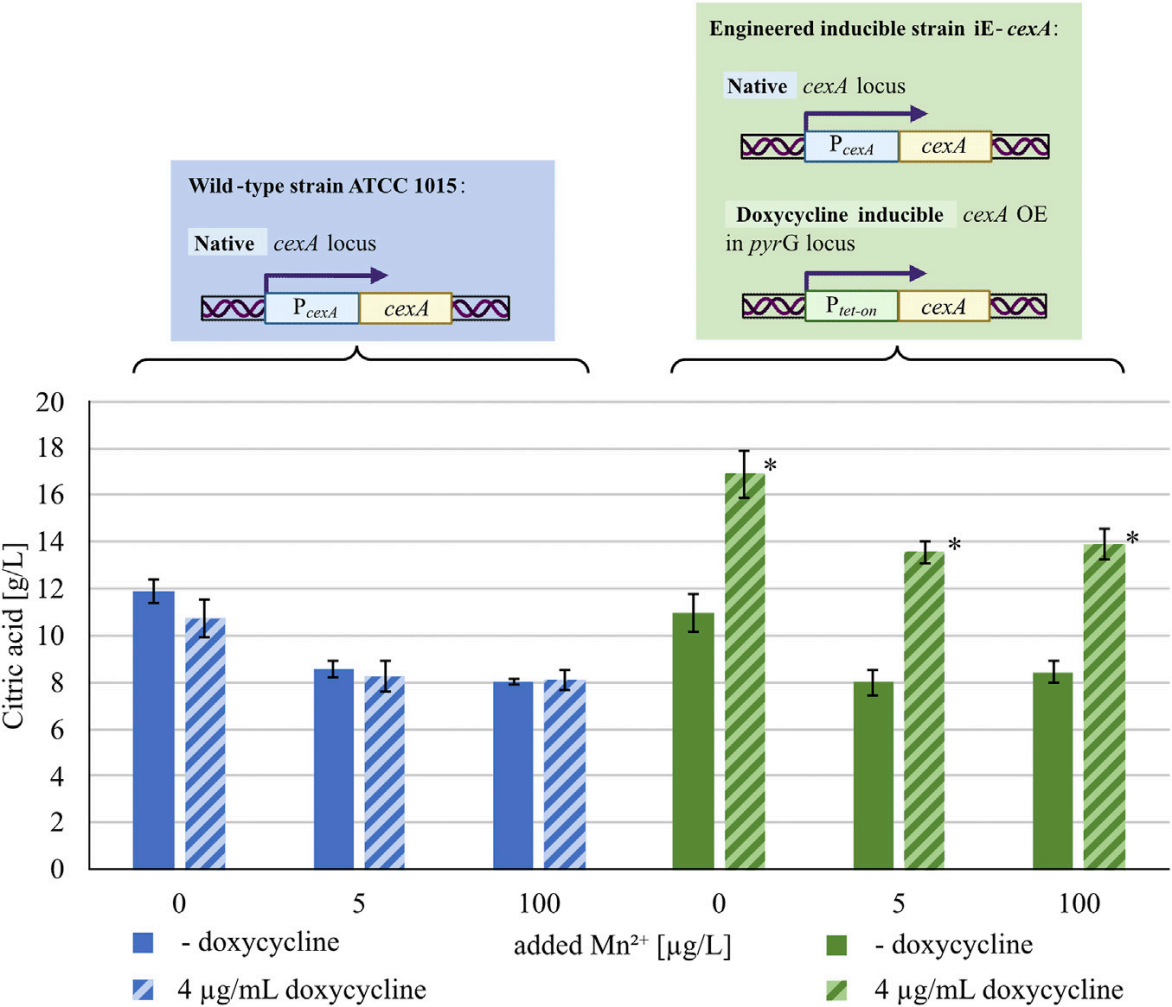
Citric acid concentrations (g/L) measured after 72 h of cultivation in MTPs. The blue/blue striped bars indicate the wild-type strain ATCC 1015 without/with 4 μg/mL doxycycline, the green/green striped bars indicate the inducible *cexA* overexpression strain iE-*cexA* without/with 4 μg/mL doxycycline. Both strains were cultivated under different manganese concentrations (x-axis). Significant differences (*p* < 0.05) determined by t-test (n = 8) are highlighted by an asterisk.

Since the repressive effect of manganese(II) ions leads to lower transcription of *cexA*, overexpression of the transporter under a manganese-insensitive promoter should restore citric acid production under manganese-sufficient conditions. To test this hypothesis, an overexpression strain of *cexA* with a doxycycline-inducible tet-on system (iE-*cexA*) was cultured at different manganese concentrations in the absence and presence of the inducer and tested for citric acid secretion after 72 h (Figure 3).

Under manganese-deficiency, the wild-type strain ATCC 1015 produced around 11–12 g/L of citric acid after 72 h of cultivation, both with and without doxycycline, showing that the addition of doxycycline did not influence citric acid production. A similar production of citric acid could be measured for the strain iE-*cexA* without induction. By supplementing the medium with manganese, the citric acid production of ATCC 1015 (with and without induction) and of iE-*cexA* without induction was reduced to around 8 g/L, confirming the expected negative effect of manganese(II) ions on the secretion. Under inducing conditions and manganese-deficient conditions, the strain iE-*cexA* produced increased amounts of citric acid (17 g/L) compared to the wild-type strain, as previously reported (Steiger et al., 2019). Additionally, this strain was able to produce significantly higher amounts of citric acid even in the presence of manganese concentrations up to 100 μg/L. With a secretion of about 14 g/L citric acid, the induced strain shows higher citrate titers compared to the wild-type strain even under manganese-deficient conditions. A manganese concentration of 100 μg/L is also exceeding a previously reported threshold of <5 μg/L, described as critical for efficient citric acid production (Fejes et al., 2020). Nevertheless, a decrease in citric acid titers from 17 to 14 g/L was observed between manganese-deficient states in the induced iE-*cexA* strain and manganese-sufficient states of either 5 or 100 μg/L. This observation can be explained by the presence of the native *cexA* locus in the genome of the overexpression strain, which is still under the control of the native *cexA* promoter. In conclusion, the results show that the transcription of *cexA* is suppressed in the presence of manganese(II) ions. This effect can be reversed upon expression of *cexA* by a manganese-insensitive promoter.

**FIGURE 4:**
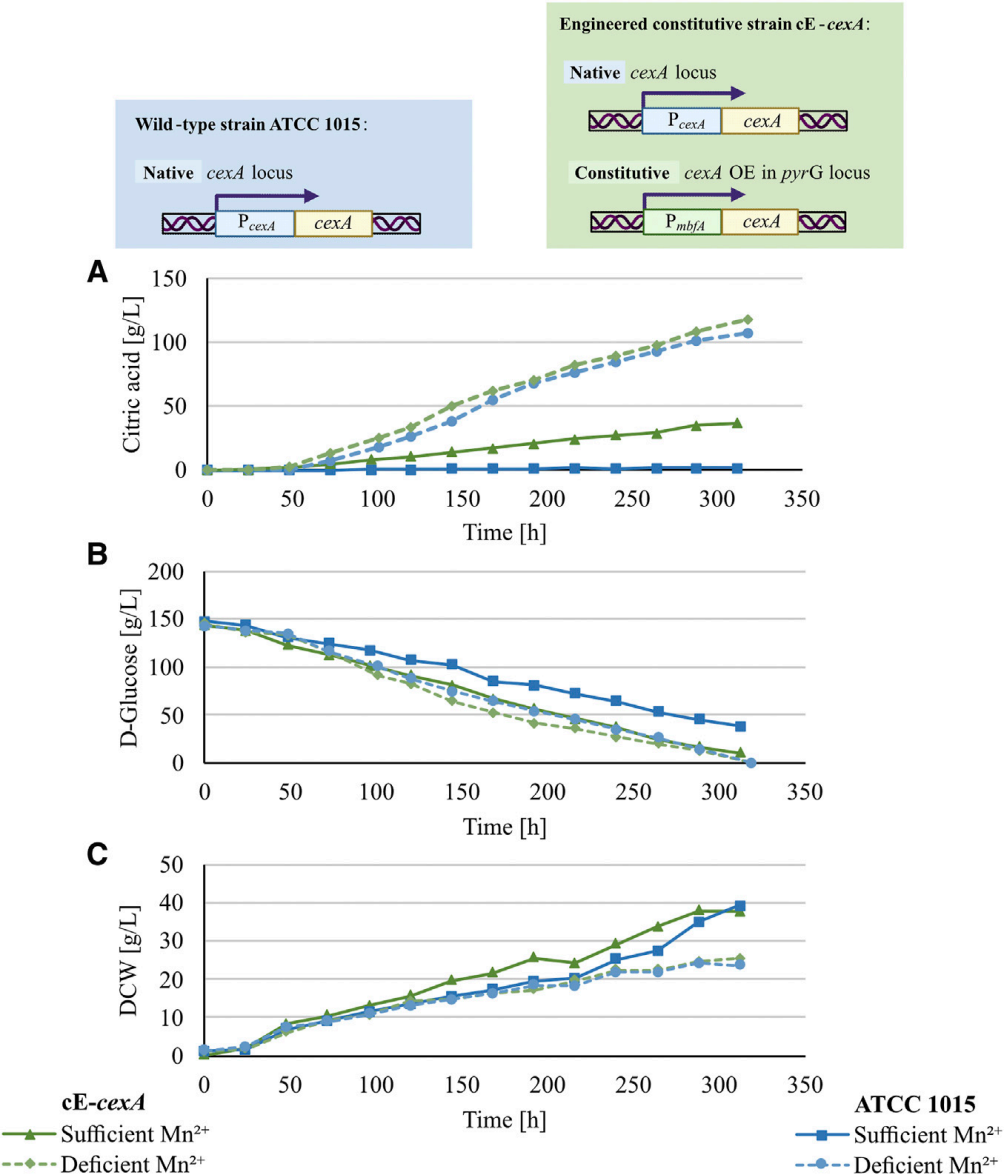
Citric acid production **(A)**, glucose consumption **(B)**, and biomass formation **(C)** of 5 L bioreactor cultivations of ATCC 1015 (blue lines) and cE-*cexA* (purple lines) under sufficient- (continuous lines) and deficient- (dotted lines) manganese concentration.

In an industrial setting, the addition of an inducer such as doxycycline is not preferred. Thus, to test an alternative expression system for CexA, a constitutive *cexA* overexpression strain, cE-*cexA,* was tested in a 5 L-bioreactor cultivation under manganese-deficient and -sufficient conditions and compared to strain ATCC 1015. In this strain, a second copy of *cexA* is under the control of the p*mbfA* promoter (Blumhoff et al., 2013). Figure 4 shows the citric acid production (A), glucose consumption (B), and biomass formation (C) of both strains under the tested conditions, and the yield, molar yield, productivity, and specific productivity of the bioreactor cultivations are reported in Table 3.

**TABLE 3:** Summary of calculated cultivation parameters.

Condition	Strain	Yield [mg_citric acid_/g_glucose_]	Molar yield of citric acid/glucose [%]	Productivity [mg_citric acid_/L*h]	Specific productivity [mg_citric acid_/g_DCW_*h]
Manganese sufficiency	ATCC 1015	14	1.3	4.8	0.1
	cE-*cexA*	275	25.8	118	3
Manganese deficiency	ATCC 1015	749	70.3	337	14
	cE-*cexA*	808	75.8	370	15

As expected, the wild-type strain produced only 1.5 g/L of citric acid under manganese-sufficient conditions and 107.2 g/L under manganese-deficient conditions (Figure 4A). In comparison, the overexpression strain cE-*cexA* reached titers of 36.8 g/L under manganese-sufficient conditions and 117.7 g/L citric acid under manganese-deficient conditions (Figure 4A). For both strains, biomass titers were higher under ` conditions compared to deficient conditions reaching 23.5 g/L for ATCC 1015 and 25.2 g/L for cE-*cexA*. This is in agreement with the literature describing that manganese is an essential component for sufficient growth and biomass formation (Kisser et al., 1980). While the wild-type strain could only produce 1.5 g/L citric acid under manganese-sufficient conditions, the *cexA* overexpression strain was able to achieve an approximately 25-fold higher titer with 36.8 g/L. However, the titer was still lower compared to the manganese-deficient conditions showing that the expression by p*mbfA* is not strong enough to fully replenish transcription to the level of the native promoter of *cexA* under manganese-deficient conditions. Despite being described as a strong promoter (Blumhoff et al., 2013), *mbfA* expression was found to be only moderate under citric acid producing conditions (Lu et al., 2022). Α single-point transcript measurement of *cexA* after 72 h in the bioreactor revealed a moderate 2.4-fold upregulation under manganese-deficient and 5.5-fold upregulation under manganese-sufficient conditions, comparing cE-*cexA* to ATCC 1015. Therefore, it can be hypothesized that further overexpression and deregulation from the native *cexA* promoter would lead to even higher citric acid titers under manganese-sufficient conditions.

It should be noted that under manganese-sufficient conditions, glucose was almost completely consumed and only 38.5 g/L remained in the ATCC 1015 and 10.4 g/L in the cE-*cexA* cultures. This shows that despite a higher biomass concentration most of the glucose is respired to CO_2_ under manganese(II)-sufficient conditions, which is also reflected in the significantly lower dissolved oxygen (DO) levels in the bioreactor.

The results obtained in the bioreactor cultivations (Figure 4) are in agreement with the results obtained in the MTP cultivation (Figure 3): by expressing CexA from a non-native promoter, such as pTet-On (in strain iE-*cexA*) or *mbfA* (in strain cE-*cexA*), the suppressive effect of manganese ions can be counteracted. Overall, a regulatory effect of manganese(II) ions on *cexA* at the transcriptional level was confirmed. There needs to be further investigation into how manganese affects *cexA* transcription directly, for example, by altering the activity of transcription factors, or indirectly, for example, by inhibiting manganese-dependent superoxide dismutase (Del Valle and Scheckhuber, 2022) causing oxidative stress. Transcriptional factors that are redox-sensitive may regulate *cexA* in this way (Cimino et al., 1997; Mendoza-Martínez et al., 2020; Pérez-Pérez et al., 2022). Reported transcriptional regulators of *cexA* are LaeA, InuR, and AmyR. The control mechanism attributed to LaeA, a methyl-transferase, is centered on its ability to modify the methylation levels of histones H3K4 and H3K9, thus regulating the transcription of *cexA* (Kadooka et al., 2020). InuR is a Zn(II)_2_Cys_6_ transcriptional activator. When deleting the *inuR* gene, *cexA* is downregulated 9.6-fold in comparison to the expression in *A. niger* N402 and a putative InuR binding side was predicted in the promoter of *cexA* (Yuan et al., 2008). Furthermore, AmyR, the transcriptional regulator of starch degradation, has an impact on *cexA* transcription. In a Δ*amyR* strain *cexA* is 54-fold downregulated on maltose compared to the *A. niger* N402 parental strain (GSE98572; Gruben et al., 2017). This is in line with a proposed AmyR influence on citric acid production (Hu et al., 2017). Additionally, we could identify a binding site for AmyR (CGG-N_8_-CGG site (Petersen et al., 1999), −388 bp from translational start site) within the native promoter of *cexA*, supporting a possible regulatory function. To obtain further insights into the relationship between CexA and the presence of manganese(II) ions, we focused on morphology, which is known to be significantly changed under manganese-deficient conditions.

### 3.3 In the presence of CexA, a more branched phenotype can be observed

**FIGURE 5:**
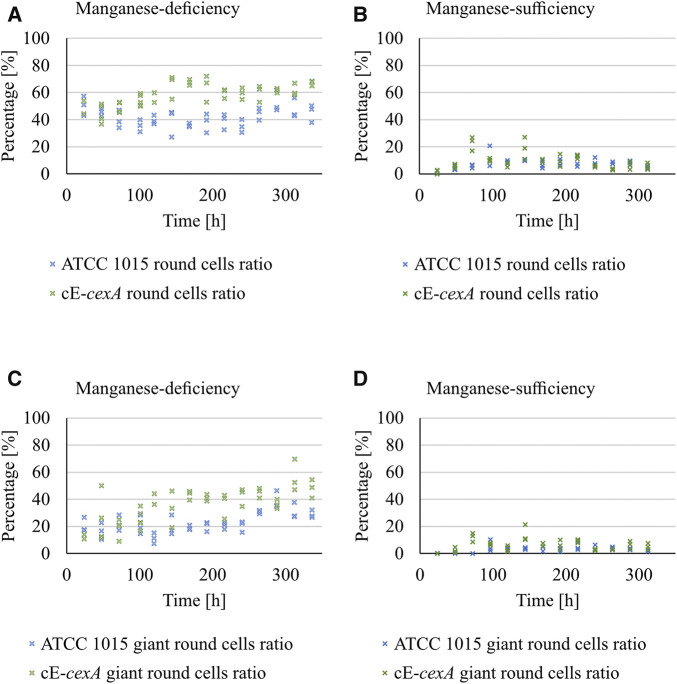
Ratio of the amount of round cells (including the giant round cells) **(A,B)** and of the giant round cells only **(C,D)** over the total amount of cells is shown in percentage for ATCC 1015 and cE-*cexA* under manganese-deficiency **(A, C)** and–sufficiency **(B, D)**.

First, the morphology of ATCC 1015 and cE-*cexA* was monitored during bioreactor cultivation (Figure 5). Round cells and giant round cells derived from the shearing of swollen hyphae were analyzed and taken as a measure for good citric acid production morphology. The ratio of the amount of round cells (including the giant round cells) and of the giant round cells only over the total amount of cells is shown in percentage in Figure 5.

Under manganese-deficiency, the cE-*cexA* strain shows an overall higher ratio of round and giant round cells (0.59 and 0.36) compared to ATCC 1015 (0.42 and 0.23). Under manganese-sufficient conditions this difference is less pronounced (0.09 and 0.06 for cE-*cexA*; 0.07 and 0.03 for ATCC 1015). However, the expression strength of the p*mbfA* promoter might not be strong enough, thus leading to a less pronounced difference in manganese-sufficiency in comparison to the manganese-deficient condition.

To test whether CexA actively contributes to the citric acid production morphology, further microscopy analyses were performed on a micro-morphology level for strains having different levels of CexA. The parental ATCC 1015 strain, the CexA inducible iE-*cexA* strain, and the loss of function mutant of CexA - D-*cexA*. The number of hyphae/germlings, lateral branches, and apical branches was counted for each strain (Table 4). While no significant difference in the number of hyphae outgrown from the ATCC 1015 conidia could be observed between manganese-deficiency and -sufficiency, the number of lateral branches was significantly increased in manganese-deficiency compared to manganese-sufficiency (from 0.16 to 2.18). Apical branching was only observed when the strain was cultivated in manganese-deficient conditions. These changes are likely to be beneficial for pellet formation and citric acid production as an increased branching leads to an increased surface area, which in turn enables more CexA to be embedded in the membrane and thus leads to increased citric acid production. First, the effect of CexA on morphology was investigated by cultivating a *cexA* knock-out mutant (D-*cexA*). In the absence of CexA, the number of lateral and apical branches in manganese-deficient conditions is significantly lower than in the wild-type strain. On average, the D-*cexA* strain formed 1.2 lateral and 1.04 apical branches, whereas 2.18 and 1.8 were counted for the wild-type, respectively. On the contrary, the *cexA* inducible strain iE-*cexA* showed both a significant increase of hyphae per germling and of lateral branches in manganese-sufficient conditions compared to the wild-type, independent from the presence of induction by doxycycline, which might be an effect caused by the presence of the transactivator of the *tet-on* promoter (Meyer et al., 2011). However, some apical branches were observed for an iE-*cexA* strain upon induction on manganese-sufficient conditions. Citric acid production conditions significantly affect fungal morphology, as previously shown. Here, we show that CexA contributes to this morphology-modulating effect by increasing the number of branches.

**TABLE 4:** Summary of microscopic analysis and quantification of morphological parameters. For each condition images of 50 germlings were used for the analysis.

Condition	Strain	No. hyphae/germling	No. lateral branches	No. apical branches
Manganese-deficiency	D-*cexA*	2.02	1.2^3^	1.04^7^
	ATCC 1015	2.16	2.18^3,4^	1.8^7,8^
Manganese-sufficiency	ATCC 1015	1.98^1,2^	0.16^4,5,6^	0^8^
	iE-*cexA* not induced	2.32^1^	0.4^5^	0
	iE-*cexA* induced	2.38^2^	0.44^6^	0.06

Mean values with the same superscript number in each column are significantly different (*p* < 0.05).

## 4 Conclusion

CexA is the main citrate exporter of *A. niger*. Here we show that the transcription of its encoding gene, *cexA,* is strongly repressed in the presence of manganese(II) ions, which explains why no citric acid can be produced under high manganese concentrations. It is possible to counteract this phenomenon by placing the *cexA* coding sequence under promoters that are not affected by manganese(II) ions. This leads to citric acid accumulation even in the presence of high manganese(II) ion concentrations. The transcriptional regulators LaeA, AmyR and InuR have been reported to regulate the transcription of *cexA*, and future research approaches may investigate how manganese interacts with these transcription factors or whether there are other unknown transcriptional regulators of *cexA* that mediate manganese signaling.

Furthermore, it is shown that CexA partially contributes to the morphological changes caused by a manganese-deficiency, especially a higher rate of branching. Further research is required for a better understanding of the regulation of fungal morphology and which morphology regulators are impacted by manganese and CexA. According to the presented work, manganese inhibits the ability of *A. niger* to secrete citric acid through transcriptional regulation of *cexA*.

## Data Availability

The raw data supporting the conclusion of this article will be made available by the authors, without undue reservation.
