# Supplementary material for: Manganese(II) ions suppress the transcription of the citrate exporter encoding gene cexA in Aspergillus niger
Source: Front Bioeng Biotechnol. 2023 Nov 22;11:1292337. doi: 10.3389/fbioe.2023.1292337 (PMC10698684; doi:10.3389/fbioe.2023.1292337)
Supplement: Supplementary file 1 [file Presentation1.pdf]

Supplementary Material

Manganese(II) ions suppress the transcription of the citrate exporter encoding gene *cexA* in *Aspergillus niger*

Aline Reinfurt[1,2], Susanne Fritsche[1,2], Vivien Bíró [3,4], Alexandra Márton[3,4], Valeria Ellena[1,2], Erzsébet Fekete[3], Erzsébet Sándor[5], Levente Karaffa[3], Matthias G. Steiger[1,2*]

[1] Research Group Biochemistry, Institute of Chemical, Environmental and Bioscience Engineering, TU Wien, Vienna, Austria

[2] Austrian Centre of Industrial Biotechnology (ACIB GmbH), Muthgasse 18, Vienna, Austria

[3] Department of Biochemical Engineering, Faculty of Science & Technology, University of Debrecen, Debrecen, Hungary

[4] Juhász-Nagy Pál Doctoral School of Biology and Environmental Sciences, University of Debrecen, Debrecen, Hungary

[5] Institute of Food Science, Faculty of Agricultural and Food Science and Environmental Management, University of Debrecen, Debrecen, Hungary

*** Correspondence:**
Matthias G. Steiger
matthias.steiger@tuwien.ac.at

A. niger ATCC 1015, manganese(II)-sufficiency

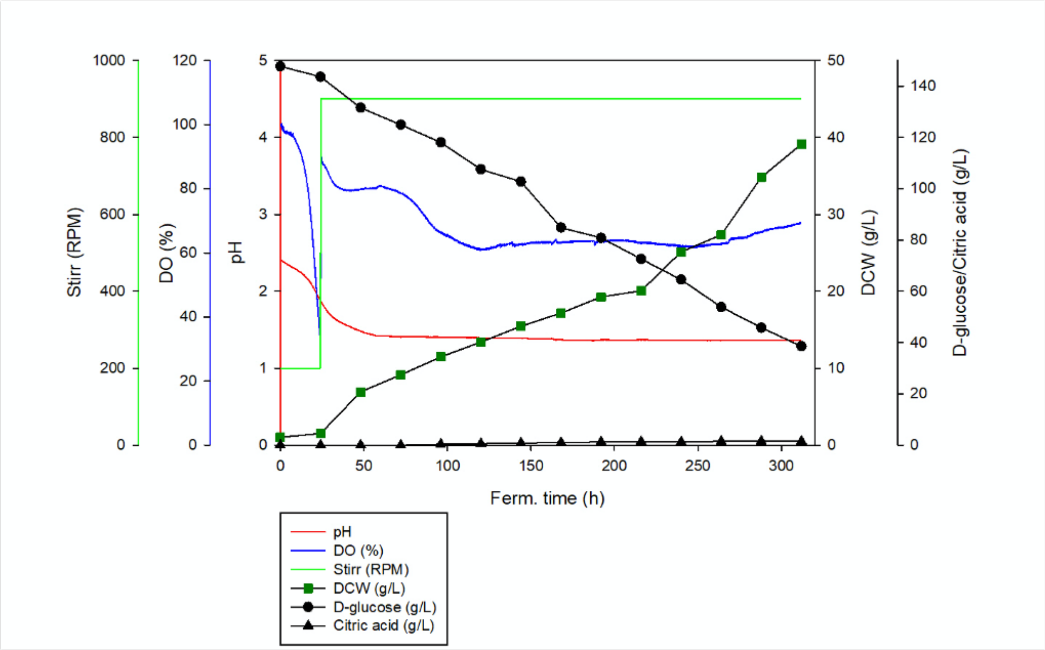

Supplementary Figure 1. Bioreactor cultivation parameters over fermentation time for *A. niger* ATCC 1015 in manganese-sufficient conditions.

A. niger ATCC 1015, manganese(II)-deficiency

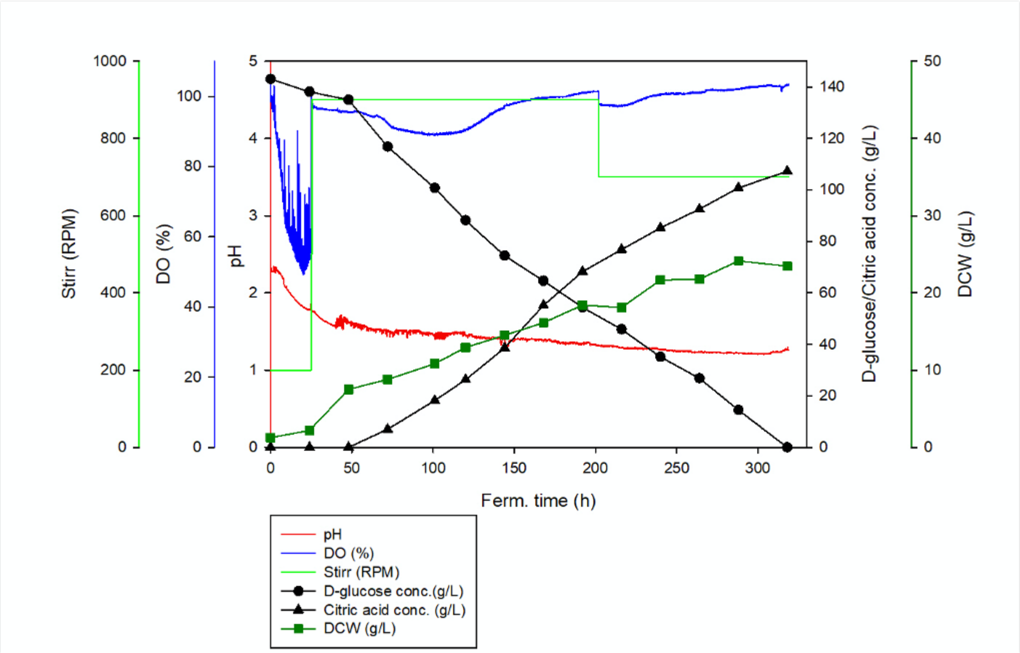

Supplementary Figure 2. Bioreactor cultivation parameters over fermentation time for *A. niger* ATCC 1015 in manganese-deficient conditions.

A. niger cE-*cexA*, manganese(II)-sufficiency

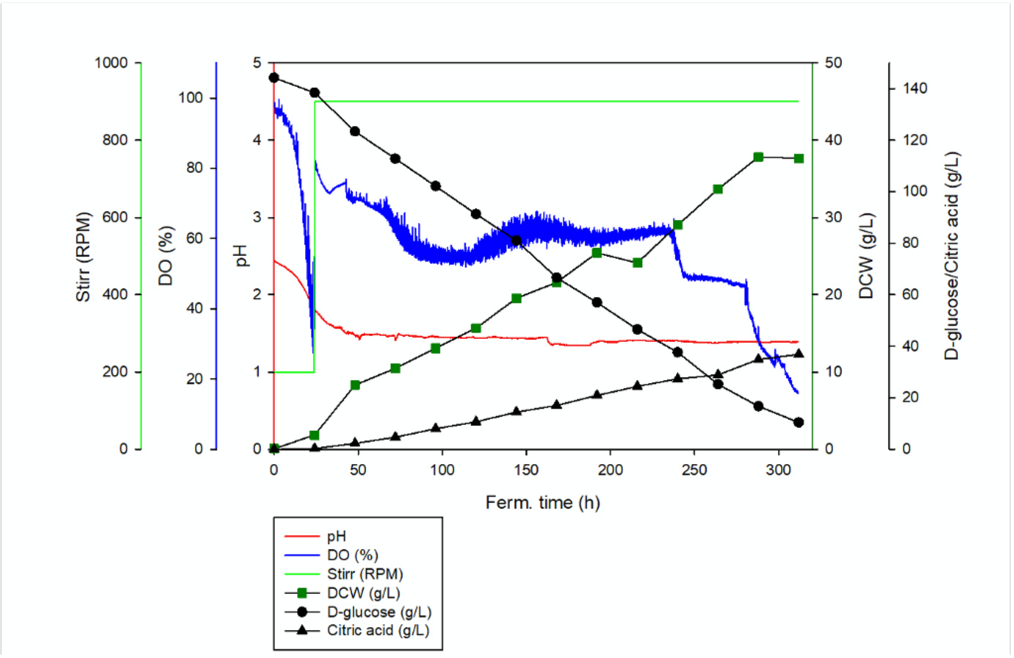

Supplementary Figure 3. Bioreactor cultivation parameters over fermentation time for *A. niger* cE-*cexA* in manganese-sufficient conditions.

A. niger cE-*cexA*, manganese(II)-deficiency

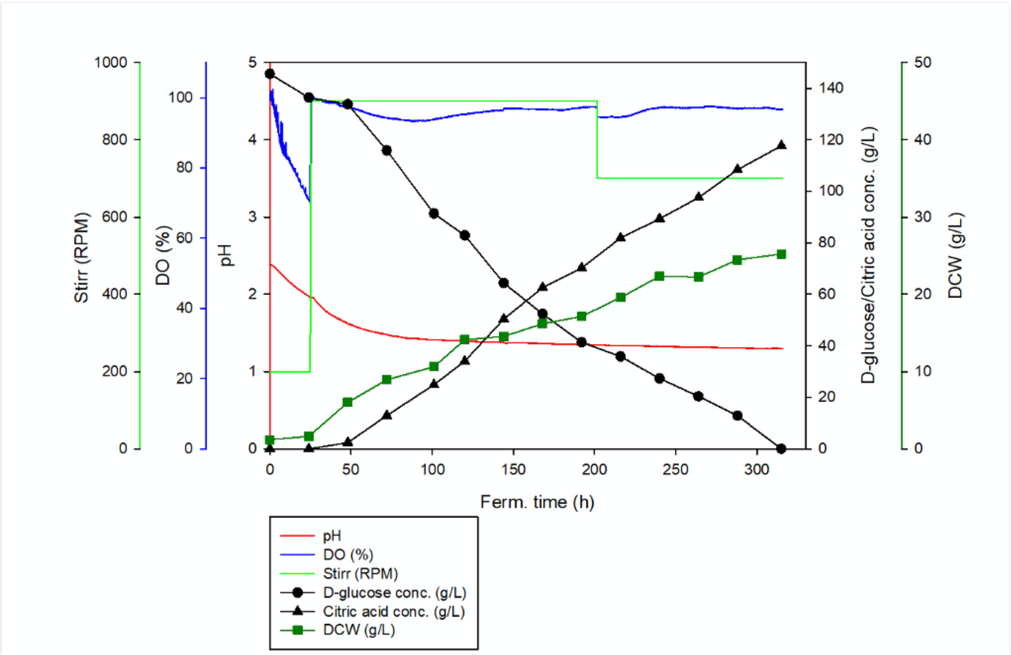

Supplementary Figure 4. Bioreactor cultivation parameters over fermentation time for *A. niger* cE-*cexA* in manganese-deficient conditions.

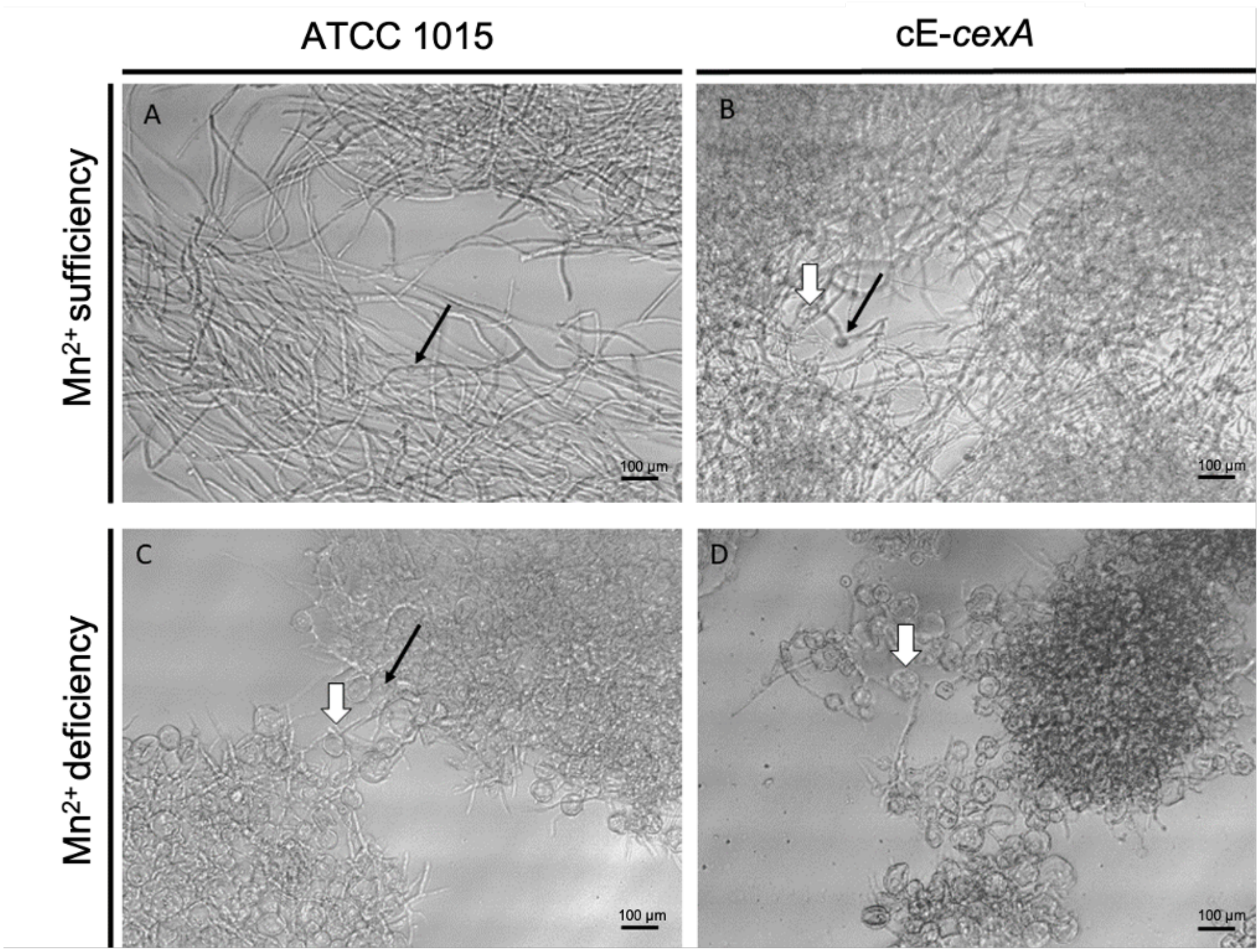

Supplementary Figure 5. Examples of round cells (black arrows) and giant round cells (white arrows) under manganese sufficiency and deficiency used for morphological analysis.
